# Exploring Oral and Vaginal Probiotic Solutions for Women’s Health from Puberty to Menopause: A Narrative Review

**DOI:** 10.3390/microorganisms12081614

**Published:** 2024-08-07

**Authors:** Marcello Romeo, Fabiana D’Urso, Giulia Ciccarese, Francesca Di Gaudio, Francesco Broccolo

**Affiliations:** 1 Department of Biology and Biotechnology, University of Pavia, 27100 Pavia, Italy; drmarcelloromeo@gmail.com; 2 Department of Experimental Medicine (DiMeS), University of Salento, 73100 Lecce, Italy; fabianadurso@gmail.com; 3 Section of Dermatology, Department of Medical and Surgical Sciences, University of Foggia, 71122 Foggia, Italy; giulia.ciccarese@unifg.it; 4 PROMISE, University of Palermo, Piazza delle Cliniche, 2, 90127 Palermo, Italy; francesca.digaudio@unipa.it; 5 Azienda Ospedaliera Ospedali Riuniti Villa Sofia Cervello, Chromatography and Mass Spectrometry Section, Quality Control and Chemical Risk (CQRC), Via del Vespro, 133, 90127 Palermo, Italy

**Keywords:** vaginal microbiota, *Lactobacilli*, probiotics, health, dysbiosis

## Abstract

The vaginal microbiota (VMB) plays a crucial role in women’s health from puberty to menopause. Traditional studies have focused on the microorganisms present within the vaginal environment and their roles in disease onset. However, the dynamic relationship between the VMB and its host remains underexplored. Common narratives emphasize the presence of *Lactobacilli* spp. as an indicator of vaginal health, yet this does not fully explain the occurrence of asymptomatic yet significant dysbiosis. Moreover, a wide array of bacterial types can inhabit the vaginal environment, suggesting that probiotic *Lactobacilli* could offer a natural, safe solution for balancing vaginal microbiota. This review examines the current literature on VMB, key factors affecting its composition, and the changes it undergoes during different life stages. Given the health-promoting potential of probiotics, we also examine their role in maintaining a healthy VMB and overall women’s health throughout life.

## 1. Introduction

Recent years have seen a surge in studies analyzing the composition and diversity of the vaginal microbiota (VMB) in women, identifying numerous factors that impact vaginal health, including internal and external contamination. The VMB is a complex ecosystem of bacteria, viruses, and fungi, with research primarily focusing on the bacterial and fungal components and their associations with women’s health [1,2,3]. 

Lifestyle changes can precipitate conditions like vulvovaginal candidiasis (VC) and bacterial vaginosis (BV) [4,5]. Untreated BV may lead to urinary tract infections, inflammation of the uterine tubes, infertility [6,7,8,9,10], adverse pregnancy outcomes [11,12,13], and an increased risk of sexually transmitted infections (STIs) like HIV, HPV, chlamydia, and gonorrhea [14]. Unlike the intestinal microbiota, the VMB is easier to analyze due to its lower bacterial diversity and the dominance of lactobacilli, which are crucial for maintaining vaginal health. A low abundance of lactobacilli increases the risk of various vaginal infections [15].

Antibiotic use is notably correlated with the onset of vaginal infections due to the disruption of the internal microbiota. Despite its lower bacterial diversity, the VMB’s unique characteristics, such as the dominance of vaginal lactobacilli, play a crucial role in health. These lactobacilli interact with the host’s immune system and produce lactic acid, creating an acidic environment that inhibits harmful bacteria [16]. However, defining a healthy VMB is complex, as vaginal dysbiosis can be asymptomatic, and not all *Lactobacillus* species offer equal protection. The VMB composition varies among ethnic groups and is influenced by hormonal changes [1,2,17].

Vaginal dysbiosis and related infections are common, often causing discomfort and leading to medical consultations. Conventional treatments with antibiotics and antifungal drugs are not always effective and can disrupt beneficial lactobacilli, potentially leading to recurrent BV and VC. Therefore, more effective and natural solutions are needed to balance the VMB and maintain a healthy vaginal environment [18,19].

Probiotics, particularly lactobacilli, have emerged as promising candidates for maintaining or restoring a healthy VMB post-antibiotic treatment. Systematic reviews and meta-analyses suggest that probiotics may help mitigate BV and VC, enhancing the quality of life for affected women [20]. Probiotics support the balance of the vaginal microbiota, promoting the growth of beneficial bacteria, preventing harmful microorganisms’ overgrowth, and strengthening the immune system [21].

Adopting a healthy lifestyle, maintaining proper intimate hygiene, and using probiotics can be effective complementary strategies for preventing and treating BV and VC [22]. Consulting with a specialist to select suitable probiotics and adhering to recommended dosages and treatment guidelines can significantly aid in managing these conditions.

The recent literature on VMB and its implications for women’s health has expanded significantly, with numerous reviews covering various aspects of this topic. For instance, Mei and Li [23] and Lehtoranta et al. [24] have provided comprehensive insights into the composition, diversity, and health impacts of the VMB. While these reviews have contributed valuable knowledge, our narrative review aims to fill specific gaps and offer a more nuanced exploration.

The primary objectives of this narrative review are to provide an overview of the VMB and the key factors influencing its composition, describing the main changes in the VMB during different life stages of women from puberty to menopause. Unlike previous reviews, we delve into the asymptomatic nature of vaginal dysbiosis and the diverse bacterial communities beyond *Lactobacilli* spp. Secondly, we provide a thorough examination of the impact of probiotics on the VMB, focusing on their potential to restore and maintain vaginal health through various life stages. This review not only consolidates existing findings but also introduces new perspectives on the practical applications of probiotics in women’s health.

## 2. Methods

A comprehensive literature assessment was conducted through a systematic search of the PubMed database, spanning from its inception to June 2024. The review encompassed original articles, meta-analyses, reviews, and animal studies, focusing on the intricate interplay between menopause, and probiotics. The search employed specific terms, including “vaginal microbiota” AND “probiotics” AND “menopause” OR “post-menopause” AND “gut microbiota”. The inclusion of articles in this review was meticulously determined by the authors based on their relevance to constructing a cohesive narrative review.

## 3. Understanding the Vaginal Microbiota and the Factors That Alter Its Composition

Thanks to advances in next-generation sequencing technologies and bioinformatic tools, our understanding of the vaginal microbiota community VMB, which includes bacteria, viruses, archaea, fungi and protozoa, has increased significantly. Each woman has her own unique microbial composition, which fluctuates over time and is influenced by various factors, such as diet, lifestyle, hormones, genetics, and age. Here, we focus on the bacterial and fungal members of the community, as these are the most prevalent microbes associated with vaginal health.

The composition of the vaginal microbiota in humans is relatively unique compared to that of other mammals, including non-human primates. For example, *lactobacilli* typically dominate 70 percent of the human vaginal microbiota community [4], whereas in other mammals, lactobacilli represent only a low percentage of the total vaginal microbiota composition [2]. This, in turn, is associated with low levels of glycogen and lactic acid [1]. Consequently, other mammals have a vaginal pH closer to neutrality. Advanced molecular detection methods using 16S rRNA gene sequencing have made it possible to group the human vaginal bacterial community into specific community status types (CSTs). CSTs I, II, III, and V are dominated by *Lactobacillus (L.) crispatus*, *L. gasseri*, *L. iners*, and *L. jensenii*, respectively [25,26]. Overall, CST I, II, and V are most often associated with health, whereas the predominance of CST IV may manifest clinically as BV or aerobic vaginitis [27]. Interestingly, CST III has been associated with both health and dysbiosis and often with BV.

### 3.1. Fungi and Their Role in Vaginal Microbiota

While much is known about the types of bacteria present within the vaginal environment, very little analytical attention has been reserved for fungi. These also contribute to our immune defenses, but studies analyzing their characteristics are currently very limited [28,29]. However, knowledge about the structure and distribution of fungal communities is rapidly increasing. High diversity is observed within and between individuals, but this diversity is significantly lower than that of bacterial communities and is more inhomogeneous. The fungal community, which includes yeasts and filamentous fungi that colonize the lower female reproductive tract, is referred to as the vaginal mycobiota. Vaginal fungi belong mainly to Ascomycota and Basidiomycota. The predominant fungal genera in the genitourinary tract (vagina) include *Candida*, *Cladosporium*, *Pichia*, *Aspergillus* and *Rhodotorula*. In total, approximately 390 different fungi have been associated with human skin, the vagina, the oral cavity, and intestinal samples to date [28]. The changes in bacterial communities due to dysbiosis increase the opportunities for growth in fungi and opportunistic pathogens [28]. 

From the conducted analyses, there is an evident presence of *Candida* spp. within a vaginal environment predominantly inhabited by lactobacilli, unlike environments characterized by dysbiosis. Especially in these types of vaginal environments, *C. albicans* finds fertile ground, with the consequent potential development of VC [30]. This same study [30] revealed a predominant percentage of this fungus in women who did not present any symptomatic signs and had an optimal state of health. It should also be noted that the composition of the female vaginal microbiota is defined by various factors, such as age, ethnicity, and especially by the levels and fluctuations of hormones that characterize a woman’s existence in different periods of the year. Not least is the state of the general immune system. However, these factors are not the only ones that influence the health status of the vaginal microbiota; the intake of drugs, an incorrect diet, and a lifestyle marked by stress can also alter its defensive structure. We must say that an important part is held by genetic inheritance. This is because the composition of the VMB and the type of CST vary depending on the ethnic group to which a person belongs. Therefore, while European and Asian women develop similar vaginal environments characterized by the significant presence of more lactobacilli, the same cannot be said for African American and African women, who are often more characterized by BV [31,32,33]. Hence, there are several diverse factors that define the development of a healthy vaginal microbiota and the associated bacterial capacity to thrive in acidic conditions.

### 3.2. Changes in VMB Over a Woman’s Life

#### 3.2.1. Childhood, Puberty, and Adolescence

**Figure 1:**
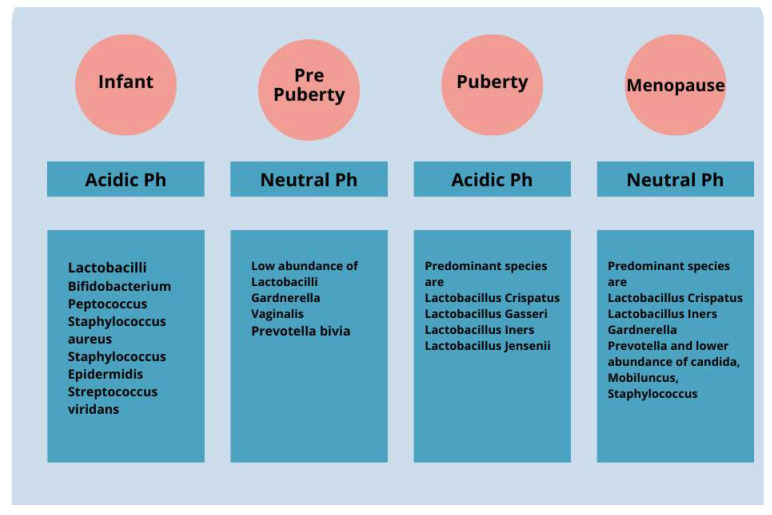
The vaginal microbiota cavity of a healthy woman changes significantly from early infancy to menopause. For instance, the predominantly diversified vaginal microbiota in children includes aerobic, Gram-positive, Gram-negative, and anaerobic bacteria. Over time, vaginal microbiota shows a decrease in diversity and becomes majorly dominated by *Lactobacillus* spp. over the pre-pubertal, adolescent, and adult phases.

Changes in the VMB over the course of a woman’s life have a significant impact on health. During childhood and puberty, there is a change in the composition of the VMB due to the influence of residual maternal estrogen (Figure 1). After birth, with the metabolization of maternal estrogen, there is a thinning of the mucosa and a change in the dominance of bacteria present. During infancy, the VMB is predominantly composed of Gram-positive, Gram-negative, and aerobic and anaerobic bacteria. The vaginal pH changes until pre-puberty and becomes neutral or slightly alkaline [34,35]. During adolescence, as estrogen levels increase, there is a thickening of the vaginal epithelium and a change in the microbiota, which becomes similar to that of adult women and is dominated by specific strains of lactobacilli [36]. The pH level of an adult healthy vagina is slightly acidic, between 3.8 and 4.5. This acidity maintains good bacterial growth while preventing bad bacterial growth.

Recent research has indicated associations between precocious puberty and the gut microbiota and its metabolites [37,38]. However, the specific mechanisms remain unclear. It is important to recognize the role of estrogen in determining VMB’s composition in pre-puberty and puberty, and this should be taken into account when designing probiotic studies in this age group to support vaginal health. 

#### 3.2.2. Importance of Estrogen

During pre-puberty and puberty, there is an increase in estrogen levels in the female body that contributes to the maturation of the genital tract and the formation of the vaginal microbiota. Estrogen promotes the production of glycogen in vaginal cells, which in turn provides a substrate for the growth of lactic acid bacteria such as *Lactobacillus*, which is known to promote a healthy vaginal environment [39]. As estrogen plays a crucial role in determining VMB composition during pre-puberty and puberty, it is important to consider this aspect when studying the effect of probiotics on these populations [39]. It is possible that the efficacy of probiotics may vary depending on estrogen levels and the VMB composition, and specific strategies may be needed to support vaginal health during these life stages. Therefore, further research is needed to better understand the interaction between estrogen, VMB, and probiotics during pre-puberty and puberty [39]. This can allow the development of more targeted and effective approaches to promote vaginal health in these critical age groups.

#### 3.2.3. Effects of Menopause on the Vaginal Microbiota

Menopause triggers significant hormonal changes, leading to alterations in the vaginal microbiota of postmenopausal women. Notably, *Lactobacillus* levels decrease postmenopause, with hormone replacement therapy potentially restoring premenopausal microbiota (Figure 1). Estrogen’s role in shaping vaginal microbiota underscores its importance, although the correlation between *Lactobacillus* levels and vulvovaginal symptoms remains uncertain. Reduced *Lactobacillus* may elevate microbial diversity and vaginal pH, heightening the risk of infection. Decreased estrogen levels during menopause can disrupt the vaginal pH balance, fostering the growth of pathogens like *Escherichia coli*, *Candida* spp., and *Gardnerella* spp., and predisposing women to BV and VC [28]. Proper intimate hygiene, diet, and timely medical intervention are crucial for prevention and management. While VC incidence declines with age due to reduced estrogen levels [39], an awareness of symptoms and early treatment remain vital across all age groups to mitigate discomfort and complications [40,41,42,43,44].

The VMB represents an intricate and multifaceted biome that directly modulates female health. This complex ecosystem plays a crucial role. Disruptions to this microbiota, particularly during the menopausal transition, are mainly caused by a lack of estrogen [39] and can lead to several deleterious conditions, such as atrophic vaginitis, recurrent urinary tract infections, and susceptibility to sexually transmitted diseases.

During the climacteric, women undergo important hormonal changes that affect several aspects of their health, including the composition of the vaginal microbiota. A decrease in estrogen leads to a reduction in the pre-dominant *Lactobacillus* spp., which are essential for maintaining vaginal health (Figure 1). This increases the risk of female health diseases.

Probiotics have emerged as a promising solution to restore and maintain the balance of the vaginal microbiota. These microorganisms provide health benefits when taken in adequate amounts, colonizing the gut and vagina and promoting the reproduction of lactobacilli. This natural strategy can be particularly helpful during menopause. Lactobacilli traverses the entire gastrointestinal tract to the rectum and, from there, migrates first to the perineum and then to the vulva before localizing at the vaginal level. The use of oral probiotics as an alternative to vaginal probiotics offers several advantages, especially for postmenopausal women [45]. Regulatory barriers and patient compliance may be easier to manage with oral probiotics, which are more widely used and have an established safety profile. Moreover, they can be easily integrated into the daily diet, improving long-term compliance among women [24]. However, further studies are needed to fully evaluate the benefits of oral probiotics on women’s health.

Limited research exists on the direct effects of probiotics on VMB and associated symptoms in menopausal women. Strategies targeting VMB health can potentially alleviate symptoms and reduce infection risk. Further investigation into probiotic interventions holds promise for managing vaginal dysbiosis in menopause and improving postmenopausal women’s quality of life.

### 3.3. Effects of Hormonal Contraceptives on the Vaginal Microbiota

Despite the widespread use of hormonal contraceptives during reproductive age, their effects on VMB and dynamics have not been fully elucidated, and there are inconsistent findings due to the various contraceptive methods used. However, there seems to be an association between oral contraceptives and a reduction in BV. For yeasts, there is no consensus on whether hormonal contraceptives increase or reduce vaginal yeast colonization or infection. A recent study showed that healthy women not using hormonal contraceptives and women using combined contraceptives had similar periodic fluctuations of VMB that correspond to the phases of the menstrual cycle and high lactobacillus abundance. In contrast, women on progestogen-only contraceptives showed altered periodic fluctuations of VMB and a low average abundance of *Lactobacillus* [46].

Regarding sexual habits, multiple sexual partners are also a known risk factor for BV/lactobacilli depletion [46]. In addition, smoking is a well-known factor to increase the risk of vaginal dysbiosis and BV, e.g., by affecting estrogen production and altering the production profile of vaginal metabolites, and increasing the levels of nicotine and its derivatives and biogenic amines [46,47].

**Figure 2:**
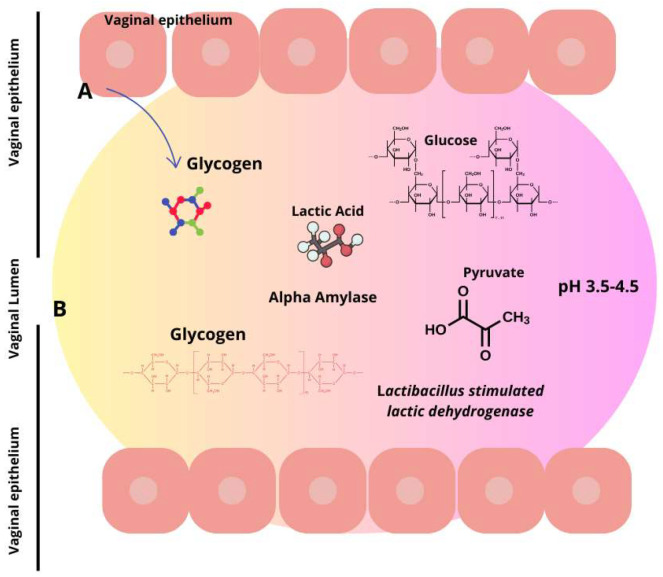
Lactic acid synthesis via glycogen breakdown in the vaginal environment. Lactic acid is produced by two distinct sources. Firstly, in the vaginal epithelium (**A**), L-lactate is produced, constituting 20% of the entire lactic acid, and secondly, from the microbiota (**B**), which contributes to the remaining 80% by metabolizing glycogen. Consequently, this produces the two isoforms of lactic acid wherein D-lactic acid is predominant.

Similarly, alcohol consumption is associated with increased cases of BV. Interestingly, new emerging research indicates that the modernization of society related to, for example, increased psychological stress, the consumption of processed foods rich in fats and carbohydrates, and urbanization has an impact on BV. Women are more stressed than men, and the effects of stress seem to extend to the vaginal tract. More specifically, research implies that chronic psychosocial stress may affect the balance of vaginal lactobacilli, potentially through immune system dysregulation and elevated cortisol levels, which further correlate with reduced vaginal glycogen, lower lactobacilli abundance, an elevated vaginal pH, and increased proinflammatory response (Figure 2).

### 3.4. Effects of Diet and Socioeconomic Factors on Vaginal Microbiota

A balanced diet can positively influence the composition of the vaginal microbiota and promote reproductive health. It is advisable to incorporate foods rich in prebiotics into one’s diet. Consuming fiber-rich foods, such as fruits, vegetables, legumes, and whole grains, can support the growth of lactobacilli. Inulin found in garlic and onions has been shown to help maintain a healthy vaginal environment [34,38,45,46]. Yogurt containing the probiotic *L. acidophilus* can also help maintain vaginal balance. Furthermore, kefir, kimchi, and kombucha are foods rich in probiotics.

Urbanization may play a role in influencing the composition of the vaginal microbiota by increasing diversity. Among socioeconomic factors, it appears that the level of education is associated with the composition of the vaginal microbiota. A recent study showed that Finnish women aged 25–45 years with a higher level of education more often had a VMB dominated by lactobacillus, particularly *L. crispatus* [48].

### 3.5. Impact of Urbanization on the Vaginal Microbiota

The immune system of the vaginal mucosa interacts with and regulates the composition of the VMB. This interaction is complex and involves various factors, such as epithelial and immune cells, antimicrobial peptides, pro/anti-inflammatory cytokines/chemokines, and secretory antibodies. Epithelial and immune cells in the cervicovaginal mucosa maintain homeostasis with the VMB and, at the same time, detect the presence of pathogens. These cells detect microbial structures via pattern recognition receptors such as Toll-like receptors, which induce the production of antimicrobial peptides and immunomodulatory cytokines/chemokines. When endogenous vaginal lactobacilli are depleted, and the community associated with BV prevails, higher levels and a different profile of inflammatory cytokine secretion are observed [49]. This association highlights an alteration in the vaginal environment, characterized by an altered immune response that may favor the development of pathological conditions [49]. Further studies are needed to fully understand how this relationship affects vaginal health and the onset of dysfunctions. 

### 3.6. Vaginal Infections and Antimicrobial Therapy

BV is the most common condition of VMB alteration in women. This condition affects approximately 38% of women of childbearing age each year. What characterizes it is an evident decrease in lactobacilli and an increase in the presence of atypical anaerobic bacteria. BV paves the way for the increased transmission of numerous sexually transmitted diseases, such as *Chlamydia trachomatis*, *Neisseria gonorrhoeae*, *Trichomonas vaginalis*, herpes simplex virus type 2 (HSV-2), human papillomavirus (HPV), and HIV [14,50,51,52,53,54,55,56,57]. Additionally, it represents a risk factor for other infections such as pelvic inflammatory disease, endometritis, and amniotic fluid infection. Antibiotics and antifungal drugs targeted against vaginal infections are the main modulators of the composition of the VMB [58]. For BV, the antibiotic regimens of first choice are metronidazole and clindamycin. These treatments have short-term cure rates of around 80% but have a recurrence rate of 50% within 6–12 months. Biofilm formation and antibiotic resistance by bacteria associated with BV, such as *G. vaginalis*, may be key factors for persistence and recurrence [59]. Similarly, *C. albicans* is effective in biofilm formation. Its hyphal (mycelial) form contributes to adherence and mucosal invasion, which are typical features of symptomatic disease. Biofilm provides increased virulence and resistance to antimicrobial agents of the host immune response, which may lead to recurrent VC and a reduced effect of antifungal treatments [28,29].

## 4. Probiotics

### 4.1. Forms of Probiotic Delivery

Probiotics act in the vaginal tract in several positive ways, producing substances such as lactic acid and hydrogen peroxide that help maintain a balanced pH. In addition, they can produce antimicrobial compounds and stimulate the immune system, counteracting the growth of harmful bacteria. By attaching to the vaginal walls, probiotics can also prevent pathogenic bacteria from settling and multiplying, thus helping to maintain a healthy bacterial balance [15,20,21,27,28,58,60,61,62,63,64,65,66,67,68,69].

Probiotics for vaginal health are available in different formats, such as dietary supplements or vaginal capsules and suppositories. While direct application at the site of action is possible with vaginal forms, probiotics taken orally must first pass through the digestive tract before they can reach the vaginal tract. Interestingly, both modes of intake appear to be effective, with even additional benefits for vaginal health through the action of the so-called “intestinal vagina”.

#### Advantages of Nanosystems in Probiotic Delivery

Probiotics can be easily destroyed by the acidic environment of the stomach. Nanosystems can protect probiotics through a physical barrier that resists acidic degradation, ensuring that more probiotics reach the intestine. Using nanosystems, it is possible to design a controlled release of probiotics. This can optimize the timing and localization of probiotic release, enhancing their effectiveness. Nanosystems can increase the bioavailability of probiotics by facilitating their absorption through biological barriers and improving their adhesion to intestinal cells. Probiotics are sensitive to various environmental conditions, such as temperature and humidity. Nanosystems can offer greater stability and shelf life, protecting probiotics from adverse conditions.

These are used to encapsulate probiotics and can be designed to release microorganisms in response to specific environmental stimuli. These are lipid vesicles that can encapsulate probiotics and provide effective protection against digestive enzymes and stomach acidity. These can be used to create three-dimensional matrices that incorporate probiotics, offering physical protection and allowing prolonged release. They are nanometric-sized emulsions that can efficiently deliver probiotics across cell membranes.

It is essential to ensure that the materials used in nanosystems are safe and biocompatible to avoid adverse reactions. Encapsulation efficiency must be optimized to ensure that a significant number of probiotics are delivered and released effectively. The regulation of nanosystems for probiotic delivery needs to be well-defined to ensure the safety and efficacy of products on the market.

### 4.2. L. Crispatus: Role in Vaginal Health

**Figure 3:**
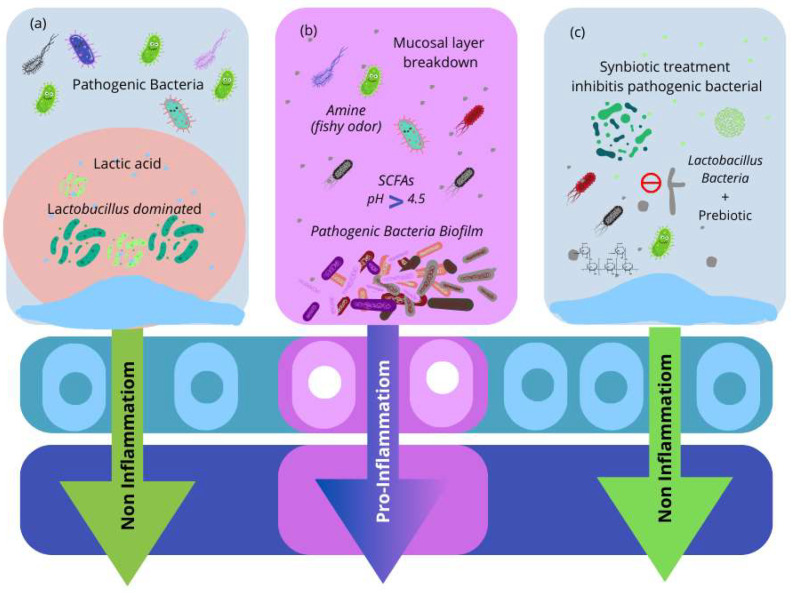
(**a**) The vaginal microbiota environment is dominated by *Lactobacillus*. *Lactobacillus* species produce lactic acid, bacteriocins, and hydrogen peroxide (H_2_O_2_), all of which have been demonstrated to defend against bacterial infections. (**b**) The microenvironment of infected microbiota. Pathogenic bacteria cause vaginal irritation and develop a biofilm on vaginal epithelial cells. Pathogenic bacteria produce short-chain fatty acids (SCFAs), which raise the vaginal pH. Furthermore, amino acid and mucosal protein catabolism form amines, a compromised mucosal layer in the vaginal tract, and inflammation. (**c**) Synbiotics are used to treat bacterial infections. Probiotic *Lactobacillus* strains, with the combination of prebiotics, result in the re-establishment of healthy vaginal microbiota.

There are a variety of *Lactobacillus* species that might be beneficial probiotics for vaginal health (Table 1) [70,71,72,73,74,75,76,77,78,79,80]. *L. crispatus* has been associated with protection against various vaginal infections, including BV and VVC. By controlling epithelial cell function, *L. crispatus* protects the epithelial barrier against inflammation and damage. *L. rhamnosus* GR-1 and *L. reuteri* RC-14 have been shown to proliferate vaginal lactobacilli burden and prevent the frequency and recurrence of BV in non-pregnant women (Table 1). These are described in Table 1. In total, 72 probiotics can be given vaginally to treat vulvovaginal infections and enable *Lactobacillus* recolonization without the necessity for transfer or concern for survival at the targeted site. In contrast to oral probiotics with long-term benefits, vaginal administration can provide direct, rapid, and targeted colonization activity to restore the altered vaginal flora (Figure 3).

*L. crispatus* is a strain of lactic acid bacteria (Figure 2) that occurs naturally in the female vaginal microbiota. However, there is a low level of evidence that probiotics, after ingestion, can really colonize the vagina. No study evidenced that *L. crispatus*, after ingestion, can colonize the vagina. This strain of bacteria plays an important role in vaginal health as it helps maintain an acidic environment that protects against infection by pathogens [29,54].

*Lactobacillus* bacteria, including *L. crispatus*, produce lactic acid that lowers the pH of the vagina, creating a hostile environment for the growth of harmful microorganisms such as *Candida (C.) albicans* or *Gardernarella vaginalis*. In addition, *L. crispatus* also produces antimicrobial substances that help prevent infections. Studies have shown that a high prevalence of *L. crispatus* in the VMB is associated with a lower incidence of vaginal infections, such as BV or VC [63,64,65,66]. Therefore, in cases where the level of *L. crispatus* in the VMB is low, e.g., following antibiotic therapy or hormonal imbalances, it may be useful to take probiotic supplements containing this bacterial strain to restore the balance of the vaginal flora and prevent infections. 

Furthermore, the presence of *L. crispatus* in the VMB has also been associated with increased fertility and a reduced incidence of complications during pregnancy, such as preterm delivery. *L. crispatus* is, therefore, a beneficial bacterial strain that plays a key role in female vaginal health [23,80,81,82]. Probiotic supplements containing this strain may be helpful in promoting vaginal health and preventing infection. 

### 4.3. Oral and Vaginal Probiotic Effects on VMB Composition

Studies evaluating the effects of probiotics on vaginal microbiota (VMB) during pregnancy have used either oral or vaginal probiotics. The oral administration of *L. rhamnosus* GR-1 and *L. reuteri* RC-14, either from 9–14 weeks of gestation until delivery [80] or for 8 weeks in mid-pregnancy [78], showed no significant impact on VMB based on the Nugent score compared to the placebo [78,80]. Conversely, Stojanović et al. [81] found that the weekly vaginal application of *L. rhamnosus* BMX 54 for 12 weeks in pregnant women from the second trimester stabilized the VMB, preventing significant changes and reducing the presence of vaginal pathogens, particularly *C. albicans*, compared to the control group. Women receiving the vaginal probiotic had a more stable VMB and did not experience significant changes during the study [81]. In contrast, women without probiotics showed a significant increase in vaginal pathogenic microorganisms, particularly *C. albicans*, over the course of the study [81].

## 5. Conclusions

Scientific studies have shown that the use of probiotics, particularly *L. crispatus* strains, can bring numerous benefits to the vaginal well-being of menopausal women [15], restoring a healthy vaginal environment and improving symptoms associated with menopause. However, the benefits obtained from probiotic supplementation are strain-specific, and therefore, the action of one strain cannot be automatically attributed to other strains, even if they belong to the same species. A probiotic strain is identified by its genus, species, and an alphanumeric designation that identifies a specific strain. In fact, while many clinical studies have relied on 16S rRNA sequencing to characterize the microbiome, its limitations in strain-level resolution and functional insights prompt a shift towards shotgun metagenomic sequencing and other advanced methodologies.

The success of a probiotic intervention depends on how well it interacts with the host’s specific factors and bacterial strains and its ability to adapt to the host’s environment and improve health. Therefore, it depends on the probiotic strain’s ability to establish and adapt to the host’s microecological niche, its ability to colonize the intestinal ecosystem, and its ability to positively influence the host’s physiological pathway and exert its health-promoting effects.

## Figures and Tables

**Table 1:** Vaginal probiotic and clinical investigations.

*Probiotic Strains*	Diseases	Key Characteristics		Ref
*L. crispatus (DSM32717 and DSM32720)*	BV	Increased vaginal lactobacilli and reduced BV-associated bacteria and VVC symptoms	Age = 18–50 years; BV (*n* = 89) and VVC (*n* = 93) patients	[70]
*L. crispatus (DSM32720*, *DSM32718 and DSM32716*	VVC			
*Lactobacillus strains*	VD	Defensive role in stabilizing and modulating vaginal microbiota	Age: 18–40 years; pre-menopausal women	[71]
*L. rhamnosus GR-1*, *L. reuteri RC-14*	BV	Restored balance of vaginal microbiota in 61.5% of patients without side effects	Age: women older than 18 years and detected with vaginal infections (*n* = 544)	[72]
*L. rhamnosus*, *L. acidophilus*, *Streptococcus thermophilus*	BV	Lowered incidence of BV and G. vaginalis recurrence after treatment	Age: 18–55 years old with recurrent history of BV (*n* = 120)	[73]
	Genital infections	Complete inhibition of pathogen proliferation as a natural alternative treatment	Age: 18–40 years; healthy, non-pregnant, pre-menopausal women	[74]
*L. rhamnosus GR-1* *L. reuteri RC-14*	GBR infection	Dramatically reduced GBS colonization rates during pregnancy	Pregnant women at 35–37 weeks of gestation with a positive GBS screening (*n* = 110)	[75]
*L. fermentum LF15* *L. plantarum LP01*	BV	Its primary function was to establish a mechanical barrier on the surface of the vaginal mucosa to prevent Gardnerella from developing. Long-term physiological defense	Age: 18–50 years; pre-menopausal, non-pregnant women (*n* = 34)	[76]
*L. fermentum LF10*, *L. fermentum LF11*, *L. acidophilus LA02*	VVC	Long-term physiological resistance by colonizing vaginal mucosa against pathogens	Age: 23–64 years; menopausal women (*n* = 30)	[77]
*(a) L. rhamnosus*, *GR-1* *(b) L. reuteri*, *RC-14*	Pre-term delivery	Supported vaginal microbiome during pregnancy	Women with less than 12 completed weeks of pregnancy (*n* = 320)	[78]
*L. rhamnosus GR-1* *L. reuteri RC-14*	BV	The vaginal microbiome was unaffected by oral probiotics administered in the early stages of pregnancy	Women between 9 and 14 weeks of pregnancy; verified by an ultrasound scan (*n* = 366)	[79]
*L. brevis CD2*, *L. salivarius subsp. salicinius*, *L. plantarum*	BV	Lactobacilli therapy resulted in a substantial decrease in IL-1 and IL-6 vaginal cytokines	Age: 20–40 years; with history of BV Non-HIV, non-pregnant (*n* = 67)	[80]
*Lactobacillus crispatus*	BV and VVC	Significantly increased lactobacilli count in the vagina while the mean proportion of some BV-related bacteria decreased. Oral and vaginal capsules lowered the amount of discharge and itching/irritation	Age: 18–50 years; BV (*n* = 89) and VVC (*n* = 93) patients	[70]

VD: vaginal dysbiosis; BV: bacterial vaginosis; VVC: vulvovaginal candidiasis; and GBR: Group B Streptococcus.

## Data Availability

The raw data supporting the conclusions of this article will be made available by the authors on request.
